# Peripheral Mechanisms of Ischemic Myalgia

**DOI:** 10.3389/fncel.2017.00419

**Published:** 2017-12-22

**Authors:** Luis F. Queme, Jessica L. Ross, Michael P. Jankowski

**Affiliations:** ^1^Department of Anesthesia, Division of Pain Management, Cincinnati Children’s Hospital Medical Center, Cincinnati, OH, United States; ^2^Department of Pediatrics, University of Cincinnati College of Medicine, Cincinnati, OH, United States

**Keywords:** muscle pain, ischemia, dorsal root ganglion, primary afferents, exercise pressor reflex

## Abstract

Musculoskeletal pain due to ischemia is present in a variety of clinical conditions including peripheral vascular disease (PVD), sickle cell disease (SCD), complex regional pain syndrome (CRPS), and even fibromyalgia (FM). The clinical features associated with deep tissue ischemia are unique because although the subjective description of pain is common to other forms of myalgia, patients with ischemic muscle pain often respond poorly to conventional analgesic therapies. Moreover, these patients also display increased cardiovascular responses to muscle contraction, which often leads to exercise intolerance or exacerbation of underlying cardiovascular conditions. This suggests that the mechanisms of myalgia development and the role of altered cardiovascular function under conditions of ischemia may be distinct compared to other injuries/diseases of the muscles. It is widely accepted that group III and IV muscle afferents play an important role in the development of pain due to ischemia. These same muscle afferents also form the sensory component of the exercise pressor reflex (EPR), which is the increase in heart rate and blood pressure (BP) experienced after muscle contraction. Studies suggest that afferent sensitization after ischemia depends on interactions between purinergic (P2X and P2Y) receptors, transient receptor potential (TRP) channels, and acid sensing ion channels (ASICs) in individual populations of peripheral sensory neurons. Specific alterations in primary afferent function through these receptor mechanisms correlate with increased pain related behaviors and altered EPRs. Recent evidence suggests that factors within the muscles during ischemic conditions including upregulation of growth factors and cytokines, and microvascular changes may be linked to the overexpression of these different receptor molecules in the dorsal root ganglia (DRG) that in turn modulate pain and sympathetic reflexes. In this review article, we will discuss the peripheral mechanisms involved in the development of ischemic myalgia and the role that primary sensory neurons play in EPR modulation.

## Introduction

Pain is a common clinical complaint resulting in a significant financial burden to both patients and society. In the U.S. alone, studies have estimated the mean cost of pain per patient at about $9K in adults and $12K in adolescents. The annual cost to society is over $635 billion (Gaskin and Richard, 2012; Groenewald et al., 2014). Because chronic muscle pain is a major cause of disability and lost productivity within the workforce (Bergman et al., 2001; Mansfield et al., 2016), the societal burden of myalgia significantly exceeds the basic expenses of pain treatment (Gaskin and Richard, 2012). Furthermore, due to the frequent underreporting and often nebulous etiology of muscle pain conditions, precise epidemiological analyses of the chronic myalgia burden are rare (Mansfield et al., 2016).

Assessment of musculoskeletal pain is also complicated by its characteristics. Unlike the typically localized pain that arises from insults to the skin, myalgia is often diffuse and more likely to evoke referred pain (Bonica, 1954; Mense and Simons, 2001; Graven-Nielsen et al., 2004). Terms used to describe sensations of deep tissue pain, such as “cramping”, “aching” and “tearing”, vary widely between patients and over time, whereas cutaneous pain tends to have a more consistent presentation often described as having a “burning” or “cutting” quality (Mense, 2008). Additionally, physicians have a particular challenge in determining appropriate pain management strategies for myalgia in that a treatment’s therapeutic efficacy is often etiology-dependent (Mense, 2008; Clauw, 2015). In the management of persistent muscle pain, first-line therapies often consist of opioids, non-steroidal anti-inflammatory drugs (NSAIDs), and physical activity regimens (Light et al., 2009; Ambrose and Golightly, 2015; Clauw, 2015; Bacurau et al., 2016); however, these types of interventions may be ineffective or even detrimental in some patient populations (Kindler et al., 2011; Murphy et al., 2011; Clauw, 2015). Thus, understanding how muscle pain arises across various diseases and injury types is paramount for increasing the availability and efficacy of specific pain management strategies.

People of all ages and demographics can be affected by muscle pain. The prevalence of the various underlying causes is known to differ between patient groups (Bergman et al., 2001; De Inocencio, 2004; Clauw, 2015; Mansfield et al., 2016). While the most frequent source of myalgia across ages is either overuse or traumatic injury (De Inocencio, 2004), there is a diversity of etiologies that include strenuous work and muscle overloading (Andersen and Gaardboe, 1993; Buckwalter, 2003), quick deceleration injuries like whiplash (Banic et al., 2004; Curatolo et al., 2004), joint diseases with peripheral inflammation (Graven-Nielsen and Mense, 2001; Kidd, 2006; Bliddal and Danneskiold-Samsoe, 2007) and ischemic injury (McDermott et al., 2004; Coderre and Bennett, 2010; Davies, 2012). This latter condition is of particular relevance because not only does it affect over 10 million people in the US alone (Norgren et al., 2007), patients often do not respond to many standard analgesic regimens for muscle pain relief (Loram et al., 2005; Clauw, 2015).

Numerous basic and clinical reports have shown that ischemic conditions are able to generate muscle pain (Alam and Smirk, 1937; Sinoway et al., 1989; Coderre et al., 2004; Laferrière et al., 2008; Ross et al., 2014). Decreased blood flow to the skeletal muscle that impairs oxygen supply sufficient to inadequately meet the metabolic demands of the tissue is a feature of multiple clinical conditions in which patients often report deep tissue pain (Dennis and Keating, 1991; Norris et al., 1993; Kasikcioglu et al., 2006; Katz et al., 2007; Nishida et al., 2009; Coderre and Bennett, 2010; McDermott, 2015). In this context, age is an important epidemiological variable. In pediatric patients, ischemic pain is often the result of pathologies like sickle cell disease (SCD), juvenile fibromyalgia (JFM) and complex regional pain syndrome (CRPS; Groeneweg et al., 2009; Zemel and Blier, 2016; Bou-Maroun et al., 2018). In adults, peripheral vascular disease (PVD) is a more prevalent cause of ischemic myalgia (McDermott et al., 2004; Norgren et al., 2007; Muir, 2009).

The origin of the muscle pain is evident in cases like PVD, where there is a mechanical obstruction of the vasculature due to atherosclerosis for example, or in SCD, in which the sickling crises induce both mechanical obstructions and hemolytic anemia (Hands et al., 1990; Beard, 2000; Meru et al., 2006; Davies, 2012; Garrison et al., 2012; Brandow et al., 2013). In other cases, anomalies in peripheral perfusion have also been hypothesized to be major contributors to the painful symptoms of conditions like CRPS and fibromyalgia (FM; Elvin et al., 2006; Coderre and Bennett, 2010; Chalaye et al., 2014). In the case of type 1 CRPS, it has been proposed that the perfusion anomalies are the consequence of a hyperactive sympathetic outflow (Bonica, 1990; Iolascon et al., 2015), usually in response to a deep tissue injury in which inflammation causes a compartment-like syndrome that impairs perfusion to the affected tissues (Coderre and Bennett, 2010). In FM, the driving factors that lead to the development of deep tissue pain are less clear, yet, studies in patients have shown impaired perfusion within the painful areas of the body (Jeschonneck et al., 2000; Morf et al., 2005; Elvin et al., 2006; McIver et al., 2006). Evidence of this deficit has been detected using enhanced ultrasound imaging of muscular blood flow during static and dynamic contractions. These studies have reported lower muscle vascularity that was accompanied by a shorter flow response to muscle activity in FM patients (Elvin et al., 2006). Furthermore, the microcirculation, measured by laser Doppler flowmetry, above sensitive points in FM patients is reported to be decreased compared to healthy controls (Jeschonneck et al., 2000).

**Figure 1:**
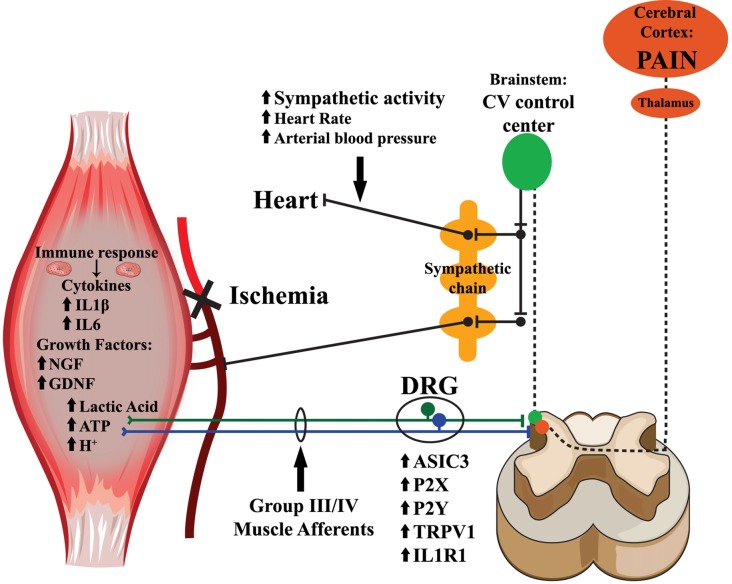
Mechanisms for muscle afferent modulation of nociception and cardiovascular reflexes after peripheral ischemia. Upon ischemic injury to the muscles, metabolites including ATP, lactic acid and protons, accumulate within the muscle interstitium. The loss of blood flow and oxygenation additionally provokes the release of growth factors and inflammatory cytokines within the injured tissue. Exposure to these endogenous substances results in the upregulation of various receptors and channels involved in sensory transduction. This leads to alterations in group III and IV muscle afferent responsiveness, particularly to metabolite stimulation. The information from the sensitized afferents is then relayed to laminae I, II, V and X of the spinal cord dorsal horn, where nociceptive signals ascend through the spinothalamic and spinobrachial tracts to the thalamus, and then further project to the cortex where they may be interpreted as painful. Group III and IV muscle afferents connecting in the spinal dorsal horn also synapse on projection neurons that ascend near the dorsolateral sulcus and activate multiple nuclei in the brainstem cardiovascular control center. In turn, these nuclei within the brainstem send descending projections to the pre-ganglionic neurons within the intermediolateral cell column of the spinal cord, and then to the paravertebral sympathetic chain ganglia, which innervate the heart and vasculature, to modulate cardiovascular responses to muscle contraction (exercise pressor reflexes; EPRs).

Severe muscle ischemia is most often not permanent. Blood flow is at least partially reestablished and this causes a complex ischemia-reperfusion (I/R) injury that is characterized by the generation of free radicals (Debold, 2015) and reactive oxygen species like hydrogen peroxide (Paradis et al., 2016) that impair mitochondrial function, damage muscle fibers and promote apoptosis (Pipinos et al., 2008a,b; McDermott, 2015; Ryan et al., 2015). In addition, during the reperfusion phase, the muscle microvasculature experiences increased permeability and injury that facilitates the sequestration of activated lymphocytes in the injured tissue. These cells, mostly macrophages and neutrophils, release pro-algesic cytokines like interleukin-1 (IL-1), tumor necrosis factor and many others (Figure 1). Intracellular granules containing radical forming enzymes can further increase cell damage and in turn enhance the immune response to injury (Blaisdell, 2002; Eisenhardt et al., 2012; Gillani et al., 2012). The duration of the insult is also relevant, as the underlying mechanisms of muscle pain generation in disorders of peripheral perfusion seem to depend on the length of ischemia and/or reperfusion; partially due to enhanced muscle atrophy and microvascular changes observed following a prolonged occlusion over those detected following a transient I/R injury (Blaisdell, 2002; Eisenhardt et al., 2012; Ross et al., 2014).

To study the basic mechanisms of post-ischemic pain, a variety of animal models have been developed. One of these, uses circumferential compression of the limb to induce an I/R-like injury (Coderre et al., 2004). Additional types of I/R, as well as prolonged ischemia, have also been modeled via surgical occlusion of a peripheral artery (Lee et al., 2005; Xing et al., 2008; Liu et al., 2010; Tsuchimochi et al., 2010; Li et al., 2014; Ross et al., 2014; Copp et al., 2015; Stone et al., 2015). Furthermore, injury-specific effects on muscle afferent sensitization and responsiveness have been investigated in both *ex vivo* (Jankowski et al., 2013) and *in vitro* conditions (Wenk and McCleskey, 2007; Light et al., 2008). These models and methods have provided a wealth of data indicating that the primary sensory afferents are likely key components in the development of ischemic myalgia (Kehl et al., 2000; Taguchi et al., 2005; Sluka et al., 2007; Gautam et al., 2010; Xu and Brennan, 2010; Ross et al., 2014).

Group III and IV primary muscle afferents are not solely involved in nociception; they also provide the peripheral sensory input for the exercise pressor reflex (EPR), a well-studied cardiovascular reflex arc that causes an increase in heart rate and blood pressure (BP) during exercise (Alam and Smirk, 1937; McCloskey and Mitchell, 1972a,b; Randich and Maixner, 1984; Zamir and Maixner, 1986; Adreani and Kaufman, 1998; Kaufman and Hayes, 2002; Hayes et al., 2008; Ives et al., 2013). Ablating these muscle sensory neurons specifically abolishes the EPR in response to muscle contraction (McCloskey and Mitchell, 1972b; Kaufman and Hayes, 2002). Animal models of ischemic injury display enhanced muscle pain responses (Issberner et al., 1996; Coderre et al., 2004; Liu et al., 2010; Seo et al., 2010; Xing et al., 2012; Ross et al., 2014), as well as altered EPR (Alam and Smirk, 1937; McCloskey and Mitchell, 1972a,b; Randich and Maixner, 1984; Zamir and Maixner, 1986; Adreani and Kaufman, 1998; Kaufman and Hayes, 2002; Hayes et al., 2008; Ives et al., 2013). This is not surprising as there is a well-documented anatomical pathway of afferent projections both directly engaging the central nociceptive networks in the ipsilateral dorsal horn and indirectly modulating the cardiovascular control centers within the medullary brainstem that influence sympathetic tone and increase systemic BP and heart rate during muscle contractions (Figure 1; Randich and Maixner, 1984; Zamir and Maixner, 1986; Kaufman and Hayes, 2002; Murphy et al., 2011). In addition, patients experiencing ischemic conditions like PVD or CRPS also have exaggerated EPRs (Adreani and Kaufman, 1998; Jänig and Baron, 2003; Li and Xing, 2012; Bartur et al., 2014; Li et al., 2014; Muller et al., 2015; Stone and Kaufman, 2015). This increased cardiovascular response to exercise can promote exercise intolerance and complicate physical therapy as well as increase the susceptibility to further cardiac events (Murphy et al., 2011; Wang et al., 2012; Gibbons et al., 2015; Bacurau et al., 2016), making this a clinically relevant complication of ischemic injury.

In this review article, we will discuss the role of peripheral afferents in sensing ischemic conditions in the periphery and the changes that injuries can induce in the response properties of these neurons. We will also examine how dynamic changes in gene expression modulate afferent responses as a direct consequence of changing external signals that include muscle metabolites, cytokines and growth factors. Finally, we will examine how the same peripheral nociceptors modulate the cardiovascular responses to exercise and how this is a potential mechanism for the development of chronic ischemic myalgia (Figure 1).

## Role of Primary Muscle Afferents in Dually Modulating Ischemic Myalgia and the Exercise Pressor Reflex

Painful sensations in the muscle are detected by group III and group IV primary afferents, which are the muscular analog of cutaneous Aδ and C fibers. These thinly myelinated (Aδ) and unmyelinated (C) neurons, whose cell bodies rest in the dorsal root ganglia (DRG), consist primarily of a long axon that gives rise to free nerve endings in the muscle tissue (Stacey, 1969; Messlinger, 1996). Some studies have also associated these free nerve endings with the muscle microvasculature and related their function with detecting specific changes in blood vessel distention (Haouzi et al., 1995, 1999; Reinert et al., 1998). While some of these muscle afferents are anatomically close to the blood vessels in muscle tissue and even express vasodilatory peptides (Molliver et al., 2005), it is known that these afferents innervate different structures in the muscle, including the perimysium, the peritendineum and even the muscle fascia (Andres et al., 1985; Messlinger, 1996; Mense, 2010; Taguchi et al., 2013). These sensory neurons also respond to a variety of stimuli including mechanical deformation of the muscles, a wide range of temperatures and changes in the intramuscular chemical environment that includes protons, various metabolites (e.g.: lactate, ATP, ADP) or in some cases noxious free radicals like hydrogen peroxide (Mense and Schmidt, 1974; Kumazawa and Mizumura, 1976; Kaufman et al., 1984; Iwamoto et al., 1985; Delliaux et al., 2009; Xu and Brennan, 2009; McCord et al., 2010; Jankowski et al., 2013; Sugiyama et al., 2017a,b).

Initially, group III and IV muscle afferents were studied to determine their roles in the generation of the EPR (McCloskey and Mitchell, 1972a; Iwamoto et al., 1985). Under normal perfusion, muscle contractions may preferentially stimulate group III afferents, but under ischemic conditions, group IV muscle afferents may be preferentially activated (Kaufman et al., 1984). Furthermore, ischemia increases the response to contractions of about 50% of group IV muscle afferents but only about 12% of group III afferents. Increased primary afferent responses under ischemia lead to increased EPRs characterized by a specific increase in the mean arterial pressure (MAP; Tsuchimochi et al., 2010). This observation is supported by similar findings in patients suffering from PVD (Baccelli et al., 1999; Li and Xing, 2012; Stone and Kaufman, 2015). The results thus suggest that there are specific subpopulations of group III and IV afferents that are sensitized by ischemia (Kaufman et al., 1984; Stone et al., 2015), and that these neurons can respond to the specific metabolites produced by muscle activity during impaired perfusion. Specifically, ATP (Kindig et al., 2007; McCord et al., 2010; Stone et al., 2014), and low pH, as consequence of increased lactic acid production (Immke and McCleskey, 2001; Molliver et al., 2005; McCord et al., 2009; Tsuchimochi et al., 2011; Pollak et al., 2014), can effectively trigger responses from muscle sensory neurons.

*In vitro* studies from Light et al. (2008) using calcium imaging on DRG neurons exposed to different concentrations of metabolites solidify this concept. Different concentrations of metabolites: pH between 7.6 and 6.2, lactate between 1 mM and 50 mM, and ATP from 300 nM to 5 μM, all replicating values observed in the muscle interstitium during mild to extreme exercise, were used to stimulate cultured DRG neurons. One of the most interesting findings is that if the metabolites were applied alone, very few neurons would be activated. However, lactate and ATP enhanced the responses induced by low pH. ATP at very high concentrations could activate neurons independently, but not at physiological concentrations. This point is supported by behavioral experiments where stimulating the muscles with ATP, lactate or low pH by themselves are unable to induce painful responses (Gregory et al., 2015). Interestingly the enhanced responses obtained by combining the metabolites in a way that it resembled physiological conditions provided effective neuronal activation that was more than additive in up to 30% of the observed neurons. Only this combination of ATP, lactate and protons was able to induce mechanical hyperalgesia (Gregory et al., 2015).

Finally, two discrete populations of chemosensitive neurons have been reported by Light et al. (2008). One population of neurons can be described as “low metabolite responders” which increases their responses from pH 7.4 (1 mM lactate and 300 nM ATP) up to pH 7.0 (15 mM lactate and 1 μM ATP). A second population of “high metabolite responders” starts responding around pH 7.0 (15 mM lactate 1 μM ATP) and increases in responses up to pH 6.6 (50 mM lactate and 5 μM ATP). These observations suggest that there is one group of primary afferents that senses the chemical environment of the muscles during normal work-related activity (metaboreceptors/“low” metabolite responders), and a separate population that detects concentrations of metabolites that are produced during noxious, ischemic contractions (metabonociceptors/“high” metabolite responders). These sensory neurons may be an important component of the sensory machinery involved in detecting ischemia and ischemic injury in muscle tissue. Studies in human volunteers support this notion. Subjects who received an intramuscular injection of the “low metabolite” mixture, reported a sensation of muscle fatigue. In contrast, when volunteers were injected with the higher concentration mixture of these metabolites, they reported a painful sensation (Pollak et al., 2014).

The different subpopulations of primary muscle afferents which include mechanoreceptors, thermoreceptors, chemoreceptors and their nociceptive variants (metaboreceptors and metabonociceptors), as well as polymodal nociceptors have been extensively characterized electrophysiologically both *in vitro* and *ex vivo* (Kaufman et al., 1984; Light et al., 2008; Jankowski et al., 2013; Ross et al., 2014, 2016; Stone et al., 2015; Queme et al., 2016). Single unit recordings using an *ex vivo* muscle/nerve/DRG/spinal cord preparation, found that about 70% of group III neurons are mechanically sensitive compared to only about 30% of group IV afferents. Most of the group IV sensory fibers (~60%) were chemosensitive. In line with the work of Light et al. (2008), two discrete populations of neurons were also observed in these studies: one responded to a “low metabolite” mixture (15 mM lactate, 1 μM ATP, pH 7.0) and one to a “high metabolite” mixture (50 mM lactate, 5 μM ATP, pH 6.6). These metabolite responsive subtypes correspond to the metaboreceptor (“low” responders) and metabonociceptor (“high” responders) populations. Moreover, the response characteristics of these neurons seem to be mutually exclusive, as very few neurons responded to both combinations of metabolites under naïve/uninjured conditions (Jankowski et al., 2013; Ross et al., 2014, 2016; Queme et al., 2016).

The two previously mentioned sub-populations of metaboreceptors (“low metabolite” responders) and metabo-nociceptors (“high metabolite” responders) along with their response properties are extensively altered following ischemic injury. Transient or prolonged ischemic insult to the muscles decreased mechanical thresholds and increased firing to mechanical stimulation in group III and IV muscle afferents (Ross et al., 2014, 2016; Queme et al., 2016). The responsiveness to “low metabolites” was also increased after I/R (Ross et al., 2014, 2016). A striking finding of these studies was that after ischemic injury, the number of metaboreceptors in the DRG was decreased compared to un-injured controls. This was concurrent with an increase in afferents responsive to both noxious and non-noxious metabolite stimulation; a population that is not readily detectable under naïve conditions (Ross et al., 2014, 2016; Queme et al., 2016). The appearance of this novel population of chemosensitive muscle afferents suggests a phenotypic switch in the composition of afferents in the DRG after injury.

The increased mechanical sensitivity in primary afferents as well as the enhanced response to “low metabolites”, combined with the greater number of afferents responding to both noxious and non-noxious metabolite stimuli, correlate with increased behavioral responses after ischemic injury. In rats, models that cause ischemia-reperfusion via a hind limb tourniquet induced mechanical hyperalgesia and allodynia in the treated animals, accompanied by cold hyperalgesia (Coderre et al., 2004). The animals in this study also showed spontaneous pain-related behaviors and contralateral pain. Moreover, this type of ischemia did not seem to induce significant nerve damage (Coderre et al., 2004), suggesting that the observed changes in behavior are not due to ischemia-induced neuropathy. Experiments using a surgical occlusion of the arterial blood flow to the upper extremities had similar findings. A model of prolonged ischemic injury using an 18–24 h occlusion of the brachial artery (BAO) induced paw guarding behaviors (a surrogate for spontaneous pain), increased mechanical hypersensitivity and decreased grip strength (Ross et al., 2014; Queme et al., 2016). I/R injury presented similar changes to the prolonged ischemic injury model, although injured animals recovered slightly faster than in the BAO model (Ross et al., 2014). In line with other animal models of pain (e.g., inflammation; Cobos et al., 2012; Grace et al., 2014), I/R also induced decreased voluntary activity (Ross et al., 2014, 2016). Altogether, these reports show the importance of primary muscle afferents in dually regulating pain and EPRs after ischemic insults to the periphery.

## Receptor Mechanisms of Muscle Sensory Neuron Sensitization after Ischemic Injury

**Figure 2:**
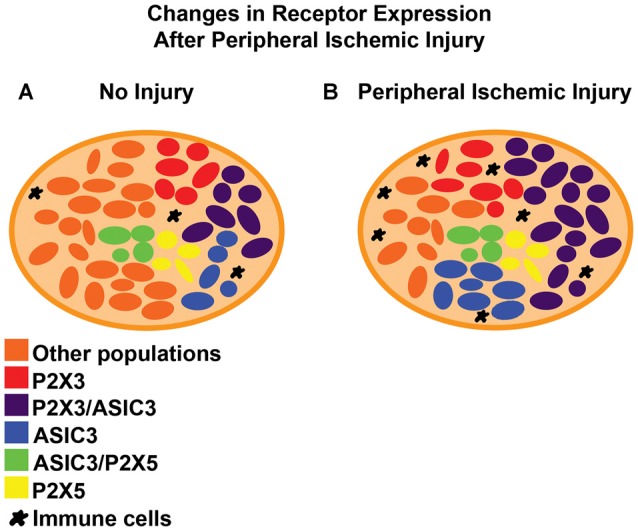
Reported changes in dorsal root ganglia (DRG) receptor expression after peripheral ischemic injury.** (A)** In uninjured/naive DRGs, receptors like ASIC3 are expressed in less than 50% of the sensory neurons and often co-expressed with different purinergic receptors like P2X3 or P2X5. Both of these receptors are reported to be expressed in more than half of the primary afferents. **(B)** After ischemic injury, there is an increase in the total number of DRG cells that are positive for ASIC3 and P2X3 (single and dual expression within neurons). Other receptors like TRPV1, P2Y1, P2X4 and ASIC1 also show increased expression after ischemic injury but the details of their distribution or co-expression after this specific injury are still unknown. Immune cells often infiltrate the tissue of the DRG in other painful conditions. Their role (along with satellite glia) in pain modulation in the context of peripheral ischemia however, has yet to be described.

After ischemic injury to the periphery, a diversity of channels and membrane receptors are upregulated in the DRGs (Figure 2). Many of these receptors have been linked with the sensitization of afferents, leading to the development of pain or modulation of the EPR. For example, the transient receptor potential (TRP) cation channel vanilloid receptor 1 (TRPV1), appears to mediate increased neuronal responses (Xing et al., 2008) and acid evoked thermal hyperalgesia (Kwon et al., 2014) in animals with a femoral artery occlusion (Seo et al., 2008). Other models show significant increases in the expression of P2X3/4/5, ASIC3 and P2Y1, which have also been linked to muscle afferent function, pain manifestation and EPR modulation post ischemia (McCord et al., 2009, 2010; Liu et al., 2010; Seo et al., 2010; Queme et al., 2016; Ross et al., 2016).

TRPV1 has been associated with the development of ischemic pain in different models. Studies in humanized hemoglobin transgenic SCD mice have shown that TRPV1 plays a role in cutaneous afferent sensitization (Hillery et al., 2011). Since SCD-induced vaso-occlusive crises primarily affect deep tissues, it’s reasonable to suggest that TRPV1 also plays a role in the development of chronic pain in SCD. A model of thrombus induced ischemic pain (TIIP) also showed that there is increased expression of TRPV1 24 h after injury (Xing et al., 2008, 2009), and DRG neurons recorded *in vitro* from these animals showed increased responses to capsaicin, a TRPV1 agonist, compared to neurons from sham animals. Also in this model, there was an increase in the sympathetic response to arterial injection of capsaicin in the animals exposed to femoral artery occlusion compared to controls. In another femoral occlusion model, the pressor response evoked by intra-arterial injection of capsaicin into the injured hind limb more than doubled the response elicited by the same injection in the contralateral, uninjured limb (Tsuchimochi et al., 2010). Collectively, this suggests a role for TRPV1 in both pain and EPR modulation post ischemia.

However, these findings are in contrast with other reports in which gene expression analysis in male mouse DRGs that innervate muscle tissue exposed to I/R or prolonged BAO injury did not show changes in TRPV1 mRNA expression 24 h after injury (Ross et al., 2014). Increased phosphorylation of TRPV1 has been suggested as a mechanism for acid evoked thermal hyperalgesia in the previously described model of TIIP (Kwon et al., 2014) and these alternative modifications may be a reason for this discrepancy. Nevertheless, the response of cultured DRG neurons to different combinations of metabolites simulating an ischemic environment is not altered by the presence of the TRPV1 antagonist JYL-1433 and are only partially blocked by the TRPV1 antagonist LJO-328 at doses that completely blocked the response to capsaicin in these neurons (Light et al., 2008). Future research on TRPV1 function in ischemic myalgia development and EPR modulation is therefore warranted.

There is ample evidence for the role of P2 receptors and how they affect afferent response to ischemia. As an example, the non-selective P2 receptor inhibitor PPADS, attenuates the EPR elicited by static contraction of the muscle (Kindig et al., 2007; McCord et al., 2010). Furthermore, usage of more specific P2X antagonists A-317491 and RO-3, had similar effects in the increased cardiovascular response induced either by static muscle contractions or by post-contraction ischemia (McCord et al., 2010). A similar effect of P2X channel antagonists on the EPR was also observed in a rat model of peripheral arterial insufficiency where blockade of the purinergic receptors prevented the enhanced EPR after ischemic injury (Stone et al., 2014). These channels also likely play an important role in modulating specific functional response properties of each afferent population. Immunocytochemical analysis of the functionally characterized DRG cells revealed that neurons responding to “low metabolites”, do not express ASIC3 while the subpopulation of neurons that is activated by noxious, pain inducing “high metabolites” usually expresses ASIC3 or TRPV1 ion channels. P2X3 appeared to be expressed in both populations (Ross et al., 2014, 2016; Queme et al., 2016).

ASIC and P2X receptors may also be key players in the afferent sensitization that is observed after ischemic injury (Dunn et al., 2001; Immke and McCleskey, 2001; North, 2002, 2004; Yagi et al., 2006; Birdsong et al., 2010; Liu et al., 2011). These channels may mediate the perception of fatigue and ischemic pain under normal conditions (Light et al., 2008; Birdsong et al., 2010). The importance of their interactions sensing the intramuscular chemical environment is highlighted by the fact that neuronal responses to low concentrations of combined ATP, lactate and low pH are blocked by low concentrations of the P2X antagonist trinitrophenyl-adenosine triphosphate (TNP-ATP). Low doses of this antagonist target only P2X5, suggesting it may play a role in sensing fatigue. At higher concentrations, TNP-ATP blocks responses to various concentrations of metabolites in the same way as the nonspecific P2 receptor antagonist PPADS (Light et al., 2008). This suggests that sensing of the more noxious “high metabolites” is dependent upon the combined action of different P2X receptors.

The role of other P2X receptors in the development of ischemic myalgia is less clear. P2X3 is upregulated after I/R injuries and the total number of positive neurons in the DRG innervating ischemia-affected muscle tissue are also increased (Cairns et al., 2003; Ross et al., 2014, 2016; Queme et al., 2016). The role of P2X3 in muscle pain development however, has been linked to inflammation. P2X3 is highly expressed in both inflammatory and post-contraction models of masseter muscle pain (Noma et al., 2013; Tariba Knežević et al., 2016). The connection to inflammatory responses is strengthened by the fact that increased expression of P2X3 and the subsequent decrease in head withdrawal thresholds observed in the post-contraction muscle pain model can be prevented by injection of interleukin 1β (IL1β) antagonists into the affected muscle. Furthermore, injection of a P2X3 selective antagonist prevents the decreased head withdrawal threshold, suggesting that the mechanical hypersensitivity is due to P2X3 activity (Noma et al., 2013). P2X3 has further been proposed to modulate the EPR enhancement resulting from ischemic injury (McCord et al., 2010). Specific P2X3 subunit knock-down using antisense oligonucleotides decreases acute inflammation-induced mechanical and thermal hyperalgesia, as well as the mechanical allodynia observed after spinal nerve ligation (Barclay et al., 2002; Honore et al., 2002; North, 2004), suggesting a role for P2X3 upregulation in the development of pain. However, these studies only assessed cutaneous pain-like behaviors. In the case of P2X4, there is an upregulation of mRNA extracted from whole DRG lysates, after I/R injury (Ross et al., 2014). Nonetheless, the expression profile of this receptor does not seem to be confined to the populations of small sensory neurons that are typically associated with nociception (Chen et al., 2016). Therefore, more research is needed to fully decipher the involvement, if any, of P2X4 on ischemic myalgia development and EPR regulation.

P2X receptors also modulate the function of ASIC3, another key mediator of pain generation. Targeting this acid-sensing ion channel can effectively reduce muscle pain in different animal models (Sluka et al., 2007; Fujii et al., 2008; Walder et al., 2011; Ross et al., 2016). Multiple mechanisms have been proposed on how changes in the function of ASIC3 can lead to increased afferent sensitization; including increased expression (Dworkin et al., 1994; Liu et al., 2010; Ross et al., 2016), enhanced membrane translocation (Queme et al., 2016) and increased synergy with other receptors (Birdsong et al., 2010), specifically purinergic receptors. The selective ASIC3 antagonist A-317567 has also been found to be effective at preventing the neuronal responses to different concentration of metabolites suggesting that sensing ischemia requires both P2X and ASIC3 activity. This interaction was later confirmed by an *in vitro* study showing that ATP enhances the response of ASIC3 to low pH. In this report, only the interaction between P2X5 and ASIC3 activation mimics the enhanced response to low pH and ATP that is observed in sensory neurons. Furthermore, about 25% of DRG neurons express P2X5 and of these neurons, about half co-express ASIC3 (Birdsong et al., 2010). While these findings are suggestive of possible modulation of ASIC3 sensitivity by P2X5, definitive confirmation in DRG neurons *in vivo* or *ex vivo* is still required.

In the specific context of ischemia, total DRG ASIC3 mRNA expression is increased in different injury models, and the total number of ASIC3 positive cells in the DRG increases (Queme et al., 2016; Ross et al., 2016). Moreover, the observed mechanical sensitization and the phenotypic switch in the metabolite response properties of DRG neurons that is observed after I/R is completely prevented (Ross et al., 2016). Both P2X receptors and ASICs are key players in the sensory component of the EPR (McCord et al., 2009, 2010). The increased cardiovascular response to exercise observed during ischemic conditions (Tsuchimochi et al., 2010) is attenuated by the individual blockade of either ASICs or P2X receptors (Tsuchimochi et al., 2011; Stone et al., 2014) suggesting a role for these channels in dually regulating both pain and sympathetic reflexes after ischemia.

P2X receptors are not the only purinergic receptors that are relevant after ischemic injuries. In a prolonged ischemia model, expression of the ADP sensitive, P2Y1 receptor, was found to be upregulated in the DRGs (Ross et al., 2014; Queme et al., 2016). Often associated with thermal hyperalgesia (Molliver et al., 2011; Jankowski et al., 2012; Queme et al., 2016), P2Y1 upregulation in the DRG was reported to modulate the increased pain related behaviors observed after ischemic insult (Queme et al., 2016). Similar results were found in the TIIP model, where blockade of P2Y1 with the selective antagonist MRS2179 prevented the induction of thermal hyperalgesia by low pH saline injections (Kwon et al., 2014). Altogether, these data suggest a diverse array of receptors and channels within group III and IV muscle afferents contribute to the development of pain and modulate the EPRs after peripheral ischemia.

## Role of Cytokines and Growth Factors in Ischemic Myalgia Development and EPR Modulation

Ischemic injury alone does not likely drive all of the aforementioned changes in primary muscle afferents. Increased gene expression and concomitant afferent sensitization can also be linked to increased signaling from the damaged muscle tissue. Current evidence points at two important sources: cytokines and growth factors. These molecules are released into the intramuscular environment in response to the tissue damage caused by ischemia (Ascer et al., 1992a,b; Seekamp et al., 1993; Sternbergh et al., 1994; Emanueli et al., 2002; Turrini et al., 2002; Ross et al., 2014, 2016). These substances can trigger painful responses and induce peripheral afferent sensitization (Oprée and Kress, 2000; Airaksinen and Saarma, 2002; Obreja et al., 2002; Amaya et al., 2004; Makowska et al., 2005; Anand et al., 2006; Binshtok et al., 2008; Svensson et al., 2008; Yang et al., 2013; Ross et al., 2016). Some of the most common factors in this context are: nerve growth factor (NGF), glial cell line derived neurotrophic factor (GDNF) and inflammatory cytokines.

NGF has been frequently linked with the development of pain and hyperalgesia in various animal models and clinical conditions (Amaya et al., 2004; Price et al., 2005; Hoheisel et al., 2007; Hayashi et al., 2011). In the context of muscle ischemia, NGF plays an important role in the repair of both vasculature and muscle tissue (Emanueli et al., 2002; Turrini et al., 2002; Karatzas et al., 2013; Diao et al., 2016). At the same time, NGF has pro-nociceptive effects through modulation of the response properties of group III and IV afferents (Hoheisel et al., 2005; Ellrich and Makowska, 2007; Svensson et al., 2008; Murase et al., 2010). NGF also seems to play a role in the development of exacerbated EPRs during ischemia. Administration of anti-NGF antibodies can prevent the increase in arterial pressure and heart rate observed during exercise in the femoral artery ligation model (Lu et al., 2012).

GDNF, another growth factor frequently tied to pain perception, is highly expressed in the muscles after ischemic injuries (Ross et al., 2014). It has been shown to induce muscle mechanical hyperalgesia after intense muscle contractions, and to potentiate afferent responses downstream of cyclo-oxygenase 2 signaling (Murase et al., 2013). Evidence of this comes from studies in male rats showing that mechanical hyperalgesia can be directly induced by intramuscular injection of GDNF. This increased response to mechanical stimulation can be reverted by administering the non-specific ASIC antagonist amiloride but not by capsazepine (Murase et al., 2014), suggesting that its sensitization effects are ASIC dependent with no involvement of TRPV1. So far, the relationship between GDNF and the EPR has not been studied. Nevertheless, previous research strongly suggests that NGF and GDNF signaling is an important component in the development of pain and increased cardiovascular responses after ischemic injuries.

One of the better characterized pro-nociceptive signals that is increased in injured muscles after ischemic injury is IL1β. This cytokine has been associated with pain development in multiple models ranging from muscle overuse (Noma et al., 2013; Borghi et al., 2014), to inflammation (Wang et al., 2015) and nerve injury (Gui et al., 2016). IL1β levels are increased in muscle tissue after acute intense swimming (Borghi et al., 2014), and administration of an IL1β antagonist before and 12 h after exercise prevents the development of mechanical hyperalgesia (Borghi et al., 2014). These findings point to IL1β as an important molecule in the development of muscle pain after injury. In the context of ischemia, the IL1β receptor IL1r1, is also upregulated in the DRG (Ross et al., 2014). Preventing this upregulation through nerve targeted siRNA injections can prevent the development of pain-related behaviors in I/R-affected mice. This strategy is also effective in preventing I/R-induced group III and IV muscle afferent sensitization, as well as the phenotypic switch in the metabolite response properties of these neurons (Ross et al., 2016). While the pronociceptive qualities of IL1β are very well established, the role of cytokines in the modulation of the EPR during ischemia is still under investigation. One of the cytokines frequently associated with increased primary afferent responsiveness and pain, interleukin-6 (IL-6), has been linked with increased EPR in response to muscle contractions in a femoral ligation model (Copp et al., 2015). The contribution of cytokine signaling to changes in the EPR after ischemic injury is therefore an important question for future research that needs to be addressed.

Another possible site of action for these various cytokines and growth factors is the DRG itself. Reports have highlighted the contributions of glial cells through cytokine release in models of neuropathic pain (Mika et al., 2013). The modulation of pain transmission through regulation of purinergic receptors in glia has also been described (Villa et al., 2010). Increased macrophage infiltration in DRG can be linked to the development of pain after peripheral nerve injury due to their ability to release cytokines and growth factors (Scholz and Woolf, 2007; Zhang et al., 2016). These data suggest that immune cells and resident glia in the DRG could also play a significant role in the modulation of ischemic pain. Yet, the specific function, if any, of glia or other immune cells in the context of peripheral ischemic injuries is still unknown and should be the subject of future research.

## Sex Differences in Primary Muscle Afferent Sensitization after Ischemic Injury

Chronic pain conditions are more prevalent in women (Wijnhoven et al., 2006a,b; Greenspan et al., 2007; Bartley and Fillingim, 2013). Multiple clinical and basic studies have shown that females are more sensitive to noxious stimulation, and more likely to require greater amounts of opioids relative to body weight following trauma or surgery (Mogil et al., 1993; Bell et al., 1994; Riley et al., 1998; Kalkman et al., 2003; Craft et al., 2004; Fillingim and Gear, 2004; Greenspan et al., 2007; Mogil and Bailey, 2010; Bartley and Fillingim, 2013; Sadhasivam et al., 2015). Furthermore, men and women have differing genetic predispositions to pain sensitivity (Kindler et al., 2011; Belfer et al., 2013; Wieskopf et al., 2015), which have also been documented in animal models (Mogil and Belknap, 1997; Mogil et al., 1997, 2000, 2011; LaCroix-Fralish et al., 2005; Juni et al., 2010; Belfer et al., 2013).

Recent studies have provided evidence for sex-dependent immune reactions that lead to differential brain and spinal cord sensitization mechanisms in a variety of rodent injury models (Sorge et al., 2011, 2015; Posillico et al., 2015; Doyle et al., 2017), but little is known about how these processes may affect primary muscle afferent function. One study, analyzing male and female gastrocnemius afferents, found that contrary to *in vivo* behavioral results suggesting lower mechanical thresholds in females, mechanical thresholds were found to be significantly higher in females during patch clamp recordings of retrogradely labeled afferents (Hendrich et al., 2012). Additionally, sex differences in glutamate response within the primary muscle afferents have been described in both humans and rodents (Cairns et al., 2001); however, basic studies of sex effects on group III and IV muscle afferent plasticity, particularly following ischemic insult, have been limited. Because of known sexual dimorphisms in disease severity and long-term outcomes in multiple conditions linked to ischemic myalgia (Wijnhoven et al., 2006b; Bartley and Fillingim, 2013; Gommans et al., 2015), including the increased occurance of CRPS (de Mos et al., 2007) and FM in women (Gran, 2003), female subjects should be considered for inclusion in future studies of ischemic muscle pain.

In our own investigation of female muscle afferents, we have found distinct changes in gene expression within the affected DRGs following I/R. Whereas males show a robust upregulation of ASIC3 after I/R, which corresponded with alterations in behavior and afferent sensitivity (Ross et al., 2014, 2016), ASIC3 levels in females are not affected with this type of injury (Ross, 2017), suggesting that ASIC3 may not serve a similar role in I/R-induced plasticity in females as it does in males. Additionally, TRPV1 and TRPM8 were found to be substantially increased in females, but not males, 1 day after I/R, which may relate to sex- and injury-dependent changes in thermal responsiveness in individual group III and IV muscle afferents (Ross, 2017).

Interestingly, human and animal studies have shown that females also have decreased EPRs compared to males (Ettinger et al., 1996; Schmitt and Kaufman, 2003; Ives et al., 2013). As ASIC3 and TRPV1 have both been shown to be integral to this reflex (Kaufman and Hayes, 2002; Li et al., 2008; Xing et al., 2008, 2009, 2012; Mizuno et al., 2011; Kaufman, 2012), further investigation of the sex- and injury-dependent expression of these channels is crucial to understanding the contributions of group III and IV muscle afferents to both pain sensitivity and EPRs.

## Clinical Significance

Adequate management of the multiple complications in patients with ischemic injuries presents a variety of challenges. While patients with conditions like PVD and FM experience great benefits from an active lifestyle and physical therapy (Busch et al., 2011; Castro-Sánchez et al., 2013, 2014), in many cases the first barrier to therapy adherence is the underlying pain, sometimes so limiting that can lead to an excessively sedentary lifestyle. The decreased activity level that follows has also been linked with increased cardiovascular risk in FM patients (Su et al., 2015; Acosta-Manzano et al., 2017).

SCD presents different challenges. Many patients with this condition are children and teenagers (Wilson and Nelson, 2015). Advances in therapies have significantly improved the life expectancy of these patients but with longer life spans new complications have arisen. The repeated ischemic injuries derived from repeated vaso-occlusive crises during the lifespan do not only result in acute painful events but can develop into intractable chronic pain (Peters et al., 2005). Current therapeutic strategies focus mainly in the treatment of the acute ischemic events (Yawn et al., 2014). Classically, pain in this type of conditions has been managed using different regimes of opioid analgesic therapies (Chou et al., 2009). A case can be made for the use of opioids in the acute setting during a vaso-occlusive crisis in SCD. However, long-term use of this therapeutic approach not only incurs the risk of developing dependence but may also be ineffective as a treatment of chronic pain (Peters et al., 2005; Painter and Crofford, 2013; Wilson and Nelson, 2015). Another clinically relevant issue is that sensitization of primary muscle afferents may occur in a sex-dependent manner and this may underlie the differing prevalence of chronic pain and cardiovascular dysfunction in men and women, which could have implications for preventative care and therapeutics.

## Concluding Remarks

Skeletal muscle ischemia is a strong driver of peripheral afferent sensitization, exerting robust effects through complex signaling cascades, resulting in the development of deep tissue pain and altered EPRs (Figure 1). Multiple studies in animal models have shown a strong link in the role of group III and IV muscle afferents as nociceptors and chemoreceptors. These physiological responses to ischemic injury allow tissue repair by causing changes in tissue perfusion and prevent further damage by triggering painful responses to normal stimuli. How these basic mechanisms are tied to the development of chronic pain and altered EPRs is still under investigation. Research aimed at the basic mechanisms involved in the chronification of pain or EPR function in conditions that feature skeletal muscle ischemia need to be prioritized in order to guide the development of new therapies for these patients.

## Author Contributions

LFQ and MPJ planned the manuscript. LFQ and JLR analyzed the literature and wrote the manuscript with guidance from MPJ. All authors edited, read and approved the manuscript.

## Conflict of Interest Statement

The authors declare that the research was conducted in the absence of any commercial or financial relationships that could be construed as a potential conflict of interest.
